# Impact of Dietary Flavanols on Microbiota, Immunity and Inflammation in Metabolic Diseases

**DOI:** 10.3390/nu13030850

**Published:** 2021-03-05

**Authors:** María Ángeles Martín, Sonia Ramos

**Affiliations:** 1 Department of Metabolism and Nutrition, Institute of Food Science and Technology and Nutrition (ICTAN-CSIC), Ciudad Universitaria, José Antonio Novais 10, 28040 Madrid, Spain; amartina@ictan.csic.es; 2 Spanish Biomedical Research Centre in Diabetes and Associated Metabolic Disorders (CIBERDEM), Instituto de Salud Carlos III (ISCIII), 28029 Madrid, Spain

**Keywords:** dietary flavanols, microbiota, immunity, inflammation, diabetes, obesity, metabolic syndrome

## Abstract

Flavanols are natural occurring polyphenols abundant in fruits and vegetables to which have been attributed to beneficial effects on health, and also against metabolic diseases, such as diabetes, obesity and metabolic syndrome. These positive properties have been associated to the modulation of different molecular pathways, and importantly, to the regulation of immunological reactions (pro-inflammatory cytokines, chemokines, adhesion molecules, nuclear factor-κB [NF-κB], inducible enzymes), and the activity of cells of the immune system. In addition, flavanols can modulate the composition and function of gut microbiome in a prebiotic-like manner, resulting in the positive regulation of metabolic pathways and immune responses, and reduction of low-grade chronic inflammation. Moreover, the biotransformation of flavanols by gut bacteria increases their bioavailability generating a number of metabolites with potential to affect human metabolism, including during metabolic diseases. However, the exact mechanisms by which flavanols act on the microbiota and immune system to influence health and disease remain unclear, especially in humans where these connections have been scarcely explored. This review seeks to summarize recent advances on the complex interaction of flavanols with gut microbiota, immunity and inflammation focus on metabolic diseases.

## 1. Introduction

Life style has changed in the last decades, mainly due to variations in dietary habits and an increased sedentarism. These circumstances have promoted the fast enhanced incidence of the so-called non-transmissible diseases, having special relevance for metabolic diseases, such as diabetes, obesity and metabolic syndrome. Indeed, the mentioned diseases are considered the most prevalent disorders worldwide [1]. Theses illnesses present a very different pathophysiology, but oxidative stress and inflammation are common key players involved in their development and progression [2,3,4,5,6]. Importantly, the metabolic state also influences immunity and inflammation, and it should be considered that these relations are bidirectional [7,8]. Similarly, in the metabolic diseases, there is an alteration on the two-way interaction between gut microbiota (GM) and the metabolic status, and as a result the richness and diversity of GM are changed, which leads to an impact on health (inflammation, immunity and metabolic status) [9]. In addition, microbiota and immunity are connected, being inflammation also involved in these relationship [10].

Metabolic diseases could be prevented to a certain degree through diet. In this regard, natural flavanols, which are abundant in fruits and vegetables, seem to exert beneficial effects on health, including diabetes, obesity and metabolic syndrome [11,12,13]. Mechanistically, it has been shown that flavanols seem to be able to modulate inflammation and immunity through the regulation of different pathways involving Toll-like receptors (TLRs), NOD-like receptors (NLRs), nuclear factor-κB (NF-κB), inducible enzymes, pro-inflammatory cytokines, chemokines and adhesion molecules, among other key proteins. However, the interplay among inflammation, immunity and GM is not completely understood, and the effect of dietary flavanols on these complex interactions is even less unknown. The aim of this review was to investigate in different experimental approaches (cultured cells, animal models, as well as the scarce human trials) these complicated relationships taking into account the potential beneficial role of dietary flavanols in a situation of metabolic disease.

## 2. Metabolic Diseases

Metabolic diseases (e.g., diabetes, obesity and metabolic syndrome) are the most prevalent disorders worldwide [1]. These diseases are characterized by metabolic disturbances that cause oxidative stress and organ dysfunctions. Thus, the hallmark of diabetes is the hyperglycemia, obesity results from an energy imbalance, and leads to the accumulation of fat, and metabolic syndrome is defined as a pathologic condition characterized by insulin resistance, hyperlipidemia, central obesity and hypertension [1,11,14].

The main pathophysiologic facts that lead to type 2 diabetes (T2D) are peripheral insulin resistance and final destruction of insulin producer pancreatic beta cells [3,14]. The pathogenesis of hyperglycemia and hyperlipidemia is associated to alterations in different crucial molecular pathways, such as insulin signaling pathway and lipid metabolism (lipogenesis and lipolysis), among others [3,14]. These changes are also related to an enhanced oxidative stress, which also contributes to the dysregulation of different pathways, namely polyol, hexosamine and protein kinase C (PKC) routes, as well as to an increased formation of advanced glycation end products (AGEs) [3,5,14]. Indeed, augmented oxidative stress is connected to cardiovascular complications (macrovascular complications), as well as to long-term dysfunction and failure of various organs, especially kidneys, eyes and nerves, which constitute the most common diabetic microvascular complications, namely nephropathy, retinopathy and neuropathy [3,5]. Importantly, this chronically enhanced oxidative stress status leads to a low-grade chronic inflammation in diabetic patients [2,3].

Obesity is basically provoked by an imbalance between the energy intake and expenditure, as mentioned above; this alteration leads to an enlargement of the white adipose tissue because of the accumulation of lipids, which also occurs in peripheral relevant organs [11]. This lipotoxic situation provokes a dysfunction in the adipose tissue that is linked to an altered lipid metabolism, impaired adipose tissue expandability and adipocyte hypertrophy [6]. Indeed, all these defects in the adipose tissue functions and the peripheral lipotoxicity are crucial in the beginning of the metabolic syndrome, and in the development of insulin resistance [6]. At molecular level, changes in the adipose tissue are responsible for modifications in pathways related to the lipid metabolism (lipogenesis and lipolysis), as well as in hormones (leptin, adiponectin, etc.) [6,11,15]. Moreover, an enhanced oxidative stress together with a chronic low-grade inflammation are present in obesity [5,6].

Metabolic syndrome is a collection of metabolic anomalies that occur together, and contribute to double the risk for cardiovascular diseases, as well as to increase 5-fold the risk for T2D [4]. These metabolic alterations include hypertension, dyslipidemia (elevated levels of triglycerides and reduced values of HDL-Cho), increased fasting glucose and central obesity [16]. Importantly, the presence of any three out of the five mentioned risk factors constitutes a diagnosis of metabolic syndrome [16]. The pathophysiology of this complex disease remains to be fully elucidated, and insulin resistance and obesity have been postulated as main causes [4]; therefore, a relevant number of dysregulated signaling pathways are altered and are responsible for the disease pathogenic manifestations. Importantly, inflammation constitutes another mechanism involved in the initiation and progression of the metabolic syndrome, and the participation of oxidative stress has also been pointed out [4]. Similarly, as in obesity and diabetes, the immune system is also affected in the metabolic syndrome [7,8].

All together suggests that inflammation constitutes a main and common player in the development and progression of the above mentioned prominent metabolic diseases [2,3,4,5,6]. In addition, it should be considered that the relationship between immunity and metabolism is bidirectional and comprises both the role of inflammation in the pathogenesis of metabolic disorders and the effect of the metabolic state, including the inflammatory situation on the regulation of immune cells [7,8]. Thus, immune cells contribute to perpetuate the metabolic disease and, in turn, the mentioned diseases negatively affect the immunity [7,8]. Importantly, another bidirectional relationship between microbiota and immunity, in which inflammation is also involved, has recently been reported to impact on the metabolic diseases [10]. However, it should be taken into account that these relevant metabolic diseases could be counteracted to a certain extent through the diet; indeed, different studies have reported a protective effect of diverse dietary components, such as flavanols, on the mentioned metabolic disorders. These health benefits exerted by these bioactive compounds are partially mediated by their modulatory effect on the immune system, inflammation and/or microbiota (see below). These complex relationships together with the potential beneficial role of dietary flavanols will be analyzed in the next sections in the context of the main metabolic diseases mentioned (diabetes, obesity and metabolic syndrome).

## 3. Microbiota, Immunity and Inflammation

The human gastrointestinal tract contains trillions of microorganisms (fungi, archaea, viruses, protozoans and predominantly bacteria), identified as GM, which exert a complex symbiotic interaction with the host [17]. The collection of bacteria in the gastrointestinal (GI) tract are taxonomically classified by genus, family, order and phyla, being the dominant phyla Firmicutes, Bacteroidetes, Actinobacteria, Proteobacteria, Fusobacteria and Verrucomicrobia [18]. Especially, Firmicutes (mainly *Eubacterium, Clostridium* and *Ruminococcus*) and Bacteroidetes represent 90% of the GM. Although GM composition remains highly stable in adults, there is a large variability among individuals mainly due to a multitude of environmental and lifestyle-related factors, such as antibiotic or drugs intake, physical exercise frequency or dietary habits [19].

During the last years, scientists have identified GM as a key regulator of human health and disease [18]. It plays many specific functions, including maintenance of structural integrity of the gut mucosal barrier [9], development of the immune system [20], protection against enteric pathogens [21] and production of several nutrient-derived metabolites with potential to affect human metabolic function [22]. For these reasons, the alteration of microbial composition (known as dysbiosis) may lead to a pro-inflammatory state and the development of many pathologies including, gastrointestinal illnesses, metabolic diseases and brain disorders [23,24,25,26]. Recently, the connection between the brain and gut, the so-called gut–brain axis, has been involved in regulating feeding and appetite, glucose homeostasis and gut motility [27]. Indeed, systemic and brain inflammation via intestinal dysbiosis have been associated with T2D, obesity, neurodegenerative diseases and many others high-incidenced diseases [27]. Although the exact molecular mechanisms that connect intestinal microbiota with metabolic diseases are only partly understood, it becomes progressively clear that the imbalance of GM affects immune system functionality and might induce inflammation, provoking clear effects on human metabolism.

### 3.1. Gut Microbiota, Immune System and Inflammation

To maintain the intestinal homeostasis and reduce the risk of metabolic diseases is essential both preserving the integrity of the intestinal mucosa and an optimal immune system functionality. The intestinal epithelium is composed of a single layer of intestinal epithelial cells (IECs) where adhesion molecules, namely claudin, occludin and zonula occludens (ZO) are essential proteins for reinforcing the epithelial barrier function and the maintenance of the structural integrity [28]. Moreover, mucin glycoproteins, which are secreted by epithelial goblet cells, form a mucus layer that serves as a protection to prevent a direct contact between the microbiota and the host tissue. In the intestinal mucosa, the interaction among mucus, antimicrobial peptides, immune cells and IgA, which limits the association of bacteria with IECs, prevents pathogens to cross the intestinal barrier and to induce an inflammatory response [29]. The breakdown of the intestinal barrier favors the translocation of bacterial components, including lipopolysaccharide (LPS), peptidoglycan and flagellin, initiating both innate and adaptive immune responses [30].

A close relationship between the commensal microbiota and the mucosal immune system, which allows IECs to tolerate commensal non-pathogenic microorganisms and to prevent intestinal pathogenic invasion, has been demonstrated. Pattern recognition receptors, including TLRs, are some proteins that the host uses to recognize commensal and pathogenic antigens and to transmit signals to adjacent immune cells, such as dendritic cells (DCs), macrophages and lymphocytes [31]. DCs recognize luminal antigens and present them to adaptive immune cells, such as T and B cells, which depending on the bacterial signals may trigger a physiological inflammatory response or lead to an immune tolerance. DCs secrete IL-10, which induces B cells to differentiate into secretory IgA cells and induces the growth of regulatory T cells (Treg), limiting mucosal inflammation and promoting tolerance. Moreover, DCs can induce effector T cells like T helper 2 (Th2), T helper 1 (Th1) and T helper 17 (Th17) cells. Th2 cells secrete various cytokines (IL-4, IL-5, IL-13) for the defense against helminths, whereas Th1 and Th17 cells produce pro-inflammatory interferon (IFN)-γ and tumor necrosis factor (TNF)-α and IL-17 to prevent extracellular pathogen-induced damages [32]. Importantly, an excess of these inflammatory factors is associated with local and systemic pro-inflammatory environments, which promote the development of diseases.

One of the main ways by which microbiota modulates both immune system and inflammation is through the production of diverse bacterial-derived metabolites from dietary components. Some of these immunomodulatory metabolites, including short-chain fatty acids (SCFAs), indole derivatives and polyamines continuously contribute to the maintenance of appropriated epithelial barrier and immune system function [33]. Bacterial fermentation of complex carbohydrates generates several SCFAs, mainly acetic acid, butyric acid and propionic acid that have a number of anti-inflammatory activities on the intestinal mucosa [34]. Thus, butyrate can induce the secretion of the epithelial repair cytokine IL-18 [35], and the production of mucus glycans, as well as the development of goblet cells in the colonic epithelium [36], promoting the integrity of the intestinal epithelium. More importantly, butyrate can also induce anti-inflammatory properties in macrophages and DCs through its interaction with the G-protein-coupled receptor GPR109A, promoting IL-10 production and Treg cell differentiation [37]. Likewise, acetate and propionate can also stimulate the expansion of pre-existing colonic Tregs [38]. Butyrate and propionate may also alter the epigenetic state of host cells through the inhibition of histone deacetylases enzymes (HDACs), contributing to the local restraint of the intestinal inflammatory response [39]. In addition to SCFA, dietary tryptophan is degraded by the microbiota into different indole metabolite derivatives, which have the potential to modulate the inflammatory response via aryl hydrocarbon receptor (AhR), which is a ligand-activated transcription factor widely expressed in immune and non-immune cells. In general, AhR activation increases the regulatory mechanisms mediated by IL-10, IL-22 and Tregs, as well as anti-microbial peptides, and contributes to the restoration of the epithelial integrity, resulting in a decreased cytokine production along with reduced microbial translocation and fibrosis in the gut [40]. Finally, the microbial metabolism of dietary arginine also produces immunomodulatory-derived metabolites (i.e., polyamides), which have been implicated in the reduction of proinflammatory cytokines and in the maintenance of the intestinal epithelium integrity [33].

Altogether, the close symbiotic relationship between the GM and the innate and adaptive immune responses highlights the importance of commensal bacteria and their derived metabolites for maintaining a healthy intestinal barrier, as well as for preventing the immune dysregulation and inflammation. Therefore, a disruption of this host–microbiota interaction equilibrium may increase the susceptibility to develop inflammatory and metabolic diseases.

### 3.2. Gut Microbiota and Metabolic Diseases

Different studies comparing healthy controls with obese and diabetic patients have showed alterations at genus and phylum levels in their microbiome [25]. In particular, frequencies of Actinobacteria, Firmicutes or Proteobacteria phyla increased, whereas Bacteroidetes and Verrucomicrobia phyla decreased. At the genus level, the depletion of SCFAs-producer bacteria, including *Eubacterium*, *Roseburia* and *Feacalibacterium*, is consistent with increases in *Clostridium*, *Collinsella*, *Fusobacterium*, *Lactobacillus*, *Megasphaera* and *Veillonella*. In general, in metabolic diseases GM is characterized by a lower diversity of species and increased opportunistic pathogens (i.e., *Clostridium spp*., *Bacteroides caccae*) [41], as well as a diminution in bacteria that efficiently harvest energy from the diet (mostly members of the Firmicutes phylum) [42]. Moreover, these GM changes include a decrease in beneficial and anti-inflammatory bacteria (i.e., *Faecalibacterium prausnitzii* and *Akkermansia muciniphila*) together with an enrichment in pro-inflammatory ones (mainly members of Proteobacteria phylum), which can be a likely source for the chronic state of low-level inflammation observed in metabolic diseases [43]. Interestingly, all these effects may also affect the permeability of the intestinal mucosa, allowing translocation into the bloodstream of bacterial products (i.e., LPS), which are involved in the stimulation of inflammatory cascades in the host [44].

More recently, greater attention has been paid to the role of diverse metabolites produced by bacteria that can be altered because of the microbiota dysbiosis present in metabolic diseases [45]. These bacterial-derived metabolites include SCFAs, indole derivatives, bile acids (BAs), trimethylamine N-oxide (TMAO), branched-chain amino acids (BCAAs) and imidazole propionate (IMP). All these metabolites could interact with several host sensing and signaling pathways in several organs, controlling a variety of functions in a favorable or detrimental way [46]. Particularly, SCFAs, which contribute to improve insulin sensitivity and glucose homeostasis, have been found to be altered in metabolic diseases [41,47]. Notably, the bacteria *Bifidobacterium* and *Akkermansia muciniphila*, which are well-known producers of specific SCFAs, have inversely been associated with low-grade inflammation, insulin resistance and metabolic disease [48]. Similarly, alterations of the GM could affect BA metabolism, especially by a decreased transformation of primary conjugated BAs into secondary BAs, leading to BA accumulation in the colon. BAs regulate glucose, lipid and energy metabolism in several tissues through complex and interrelated pathways, mainly including the farnesol X (FXR) and G protein-coupled membrane receptor 5 (TGR5) signaling cascade; consequently, an imbalance in BAs could intensify the metabolic disease [49]. Other important microbiota-derived metabolites, which are reduced in the metabolic diseases, are the indole derivatives produced by GM from dietary aromatic amino acids, such as tryptophan [50]. AhR signaling induces the secretion of glucagon like peptide-1 (GLP-1), an incretin hormone with a key role in glucose homeostasis and liver function. Therefore, the ineffectiveness of GM to produce tryptophan-based AhR ligands could also contribute to the outcome of metabolic dysfunction [51].

Altered GM found in metabolic disorders has also been associated with increased plasma levels of TMAO [52,53]. TMAO is generated by the microbial transformation of food components, such as carnitine, choline or lecithin, and its increased levels has been related with tissue inflammation, impaired glucose tolerance and metabolic dysfunction [53]. Likewise, dietary BCAAs, isoleucine, leucine and valine can also regulate glucose and lipid metabolisms, and altered BCAA levels have been associated with insulin resistance, and enhanced risk for metabolic diseases [54]. Interestingly, different bacteria (i.e., *Prevotella copri* and *B. vulgatus*) are potent producers of BCAAs, whereas others (i.e., *Butyrivibrio crossotus* and *Eubacterium siraeum*) are able to take up BCAAs, suggesting that microbiota dysbiosis may contribute to insulin resistance and metabolic diseases by modulating BCAAs levels [55]. More recently, IMP, a metabolite produced by GM from histidine, has emerged as an important microbial metabolite contributing to the development of insulin resistance [45] through the blockage of the insulin receptor cascade via mammalian target of rapamycin complex 1 (mTORC1) [56]. IMP levels are increased in subjects with prediabetes and T2D and, more importantly, this microbial metabolite was associated with dietary habits and altered microbial ecology [57].

Collectively, all these data suggest that the modulation of microbiota and their derived metabolites could potentially improve the inflammatory and metabolic abnormalities associated with metabolic disorders, reducing thus the incidence of common metabolic diseases. Although, microbiota composition can be influenced by numerous external factors, several studies have demonstrated the strong influence of diet and dietary patterns on the structure and function of GM and their effect on health [58]. For instance, it has been shown that different dietary fatty acids may impact on the microbiota–brain communication to significantly affect the pathophysiology of neuropsychiatric diseases [23]. Likewise, unbalanced diets (mainly Western diets) may induce dysbiosis resulting in increased gut permeability and low-grade inflammation. On the contrary, intake of plant-derived foods has been related with beneficial changes on GM composition. Plant-derived food constitute the primary source of dietary polyphenols (mainly flavonoids), natural compounds that can act as prebiotics promoting the growth of beneficial bacteria and inhibiting the development of pathogens. Altogether, it highlights the therapeutic significance of dietary intervention.

## 4. Dietary Flavanols

**Figure 1:**
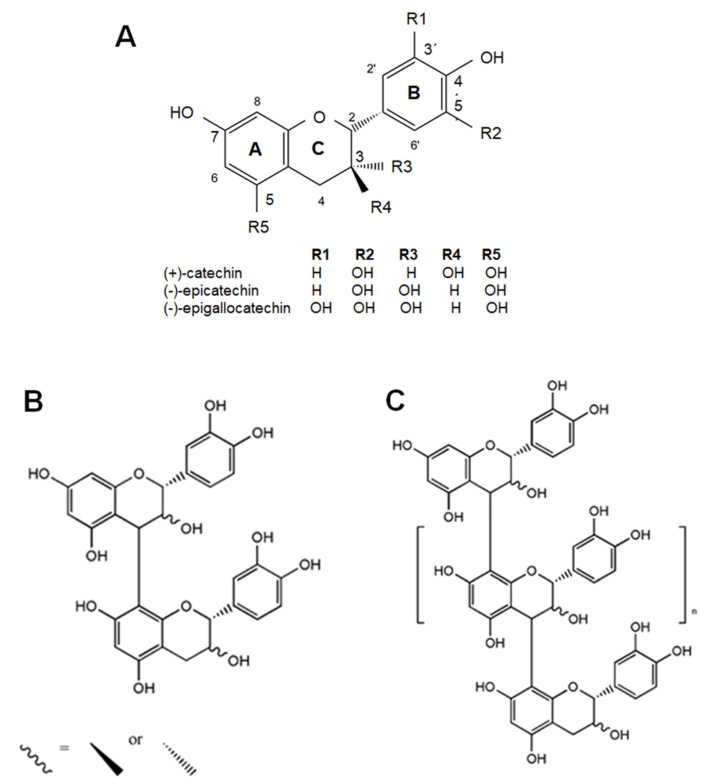
Chemical structure of main representative flavanols. (**A**) Flavanol monomers. (**B**,**C**) Procyanidins or proanthocyanidins.

Flavanols are a subclass of flavonoids, which in turn are a wide group of phenolic compounds [59]. These components are secondary metabolites ubiquitously found in plants, and significant levels of dietary flavanols have been reported in cocoa, tea, apples, grapes, berries, plum, apricots and nuts [14,59]. Flavanol molecular structure consists of two benzene rings linked by a linear three-carbon oxygenated heterocycle (C6-C3-C6) and is hydroxylated at C3 (namely, flavan-3-ols) (Figure 1) [59]. This hydroxyl group could be modified by adding a gallate group, which are especially found in tea [14,59]. Moreover, flavanols exist in food as monomers or as oligomers and polymers, and are termed catechins and proanthocyanidins (or procyanidins), respectively (Figure 1). In contrast to other classes of flavonoids, flavanols are not glycosylated in foods. Major dietary flavanol monomers include (+)-catechin, (−)-catechin and (−)-epicatechin, among others. Oligomers and polymers are defined by the type of monomer and for the kind of link among monomers. In this regard, it could be mentioned that the polymerization degree could be higher than 10 covering a wide range of molecular weights and that there are several oligomerization patterns. Importantly, some vegetables present characteristic oligomerization ways, e.g., in cocoa the monomeric units are linked through 4→8 carbon–carbon bonds forming mostly B-type dimers [13].

Upon flavanol uptake, these compounds remain stable until they reach the small intestine. Monomers are rapidly absorbed and, in the liver, they are conjugated by phase II enzymes to generate sulfates, glucuronides and methylated metabolites; however, it should be taken into account that enterohepatic recirculation could also take place with biliary elimination of the flavanol [60]. Oligomers and procyanidins are poorly absorbed in the gastrointestinal tract, and are mainly metabolized together with monomeric flavanols in the large intestine (90–95% of total intake) by the microbiota; indeed, it has been reported that gut metabolite levels in blood could be 10–100-fold higher than the parent compound [61]. These microbial metabolites peak between 9–48 h after the flavanol consumption, and are mainly phenyl-γ-valerolactones and phenolic acids, (phenylvaleric acids, m-hydroxyphenylpropionic acid, m-hydroxyphenylacetic acid and m-hydroxybenzoic acid) [60].

Understanding flavanol bioavailability and metabolism is crucial to state and explain the biological activities of these natural phenolic compounds. Numerous studies have attributed different benefits on health to flavanols, including antioxidant, anti-inflammatory, anticarcinogenic, immunomodulatory, antiallergic and antiviral effects [12,62,63,64]. Furthermore, many studies suggest an efficacy of flavanols to prevent or delay the appearance of several chronic pathologies. In this line, beneficial effects of flavanols on metabolic diseases, such as obesity, diabetes and metabolic syndrome have been reported [11,13,14,62,65]. The mechanisms that have been proposed to explain these biological actions of flavanols on the metabolic diseases are based on their capacity to act as antioxidants and to interact with numerous signaling pathways and DNA [11,13,14,62,65], although these molecular mechanisms remain to be fully elucidated. Lately, the binomial relationship between flavanols and microbiota and its impact on health and disease has also started to be considered [64,66]. Noteworthy, the impact of these natural compounds on metabolic diseases in terms of microbiota and immune system remains still largely unknown.

## 5. Effects of Dietary Flavanols on Immunity and Inflammation in Metabolic Diseases

Dietary polyphenols may modulate immune cells, cytokine production, as well as pro- and anti-inflammatory proteins in health and diseases [67]. These aspects have been supported by different works [63,67], but the connection among immunity, inflammation and metabolic diseases (diabetes, obesity and metabolic syndrome) is still scarcely studied. In this regard, during T2D it has been reported that a major flavanol in green tea, epigallocatechin gallate (EGCG), as well as epicatechin are able to prevent the inflammatory status in immune cells, which in certain cases has been associated to an alleviation of diabetic complications (Table 1 and Table 2). Thus, EGCG averted high glucose-induced inflammation in cultured aortic endothelial cells and in the aorta of diabetic mice by modulating the levels of the master inflammatory switch NF-κB (Table 1) [68]. In particular, it reverted to control values the adhesion of monocytes to vascular cells in vivo and in vitro, decreased the levels of relevant chemokines (monocyte chemotactic protein-1 [MCP-1] and the mice chemokine most closely related to IL-8, KC) by 30 and 50%, respectively, as well as adhesion molecules (intercellular adhesion molecule-1 [ICAM-1] and vascular adhesion molecule-1 [VCAM-1]); thus, ICAM-1 showed similar values to those of controls, and VCAM-1 displayed levels even lower than those of controls (150% reduction). Similarly, EGCG and epicatechin prevented the pro-inflammatory status induced by high glucose conditions in leukocytes of non-obese diabetic Goto-Kakizaki rats and in human cultured monocytes, respectively [69,70]. At molecular level, EGCG reverted to control levels the enhanced expression of inflammatory proteins (interferon [IFN]-γ, IL-1β, IL-6, IL-18) in leukocytes from GK rats, showing a reduction in their mRNA values by 82%, 62%, 77% and 76%, respectively [70]. In addition, proteins associated to the adhesion and migration of immune cells (MCP-1, integrin CD11b, S100 calcium binding protein A6 [S100A6]) were diminished by 53%, 60% and 62%, respectively in peripheral leukocytes of diabetic animals after being incubated with EGCG [70]. All these results demonstrated an improvement of the disease. Similar effects were observed in the adipose tissue of these rats, since EGCG led to significantly lower mRNA levels of IL-1β, TNF-α, IL-6, IL-18 and MCP-1 (by 68%, 71%, 78%, 49% and 57%, respectively) [71]. Remarkably, all these beneficial effects were detected at a low dose of EGCG supplementation (0.1% EGCG) and not at higher dosages (0.2 and 0.5% EGCG) [70,71]. In addition, epicatechin has also demonstrated anti-inflammatory effects against a high-glucose-induced inflammation in human cultured monocytes, as diminished the levels of pro-inflammatory cytokines (TNF-α) and NF-κB by 50% for both proteins [69]. These effects were related to epicatechin-induced epigenetic changes on histone modifications and suggested to be positive for diminishing vascular inflammation and diabetic complications.

As in diabetes, peripheral immune markers are also affected in obesity (Table 1). In peripheral blood mononuclear cells (PBMC) isolated from obese people incubated with red grape polyphenols, a reduction of the inflammatory status was detected. Thus, decreased levels of IL-1β (~70%), IL-6 (~30%) and IL-21 (an inducer of Th17 cells, 100%), and increased IL-10 production (~50%) were reported [72]. In the same line, in macrophages and adipocytes co-cultured cells incubation with a polyphenol-rich grape powder extract, oligomerized grape seed polyphenols, theaflavin-3,3′-digallate or a cocoa shell aqueous phenolic extract, as well as with some pure compounds (procyanidin B2 or epicatechin) prevented an induced pro-inflammatory challenge. Thus, a diminution in the secretion of inflammatory cytokines (TNF-α, IL-1β, IL-6, MCP-1, decreased in a range between ~30% and 80%), and chemokines (IL-8, interferon-γ-inducible protein 10, declined by 85% and 75%, respectively) was demonstrated [73,74,75,76]. Mechanistically, this diminished inflammatory capacity in macrophages exerted by flavanols to inflame adipocytes has been associated to a downregulation of the NF-κB pathway, as well as MAPKs (ERK, JNK and p38), factors such as AP-1 (c-jun) and ETS Like-1 protein (Elk-1), and oxidative stress [73,74], contributing also to improve the lipid metabolism and reduce the insulin resistance [75,76]. In addition, a switch from the inflammatory M1-like-phenotype (CD11c, CCR7, CD86) towards a less inflammatory M2-like phenotype (CD206, CD163) macrophage via adenine monophosphate activated protein kinase (AMPK) induced by flavanols has also been reported [75]. Similarly, a modulatory effect on Treg cells has been demonstrated in PBMC from obese volunteers receiving EGCG, showing an increased number of Tregs and IL-10 production (3% and 48%, respectively), together with an inhibition of NF-κB route via epigenetic changes [77].

Regarding the in vivo studies, just two human clinical interventions have been carried out. In a randomized controlled double-blind trial, the sensitivity of PBMC from obese volunteers submitted to a bacterial challenge (LPS treatment) was enhanced after incubating the cells with a dietary grape powder, as IL-1β and IL-6 secreted values increased by 17% and 14%, respectively [78]. Nevertheless, this result was associated with a beneficial effect in obese individuals, as this population is more prone to develop infections. Likewise, in another interventional human study, a beneficial effect on health and longevity was demonstrated by green tea supplementation through the elongation of leukocyte telomeres in obese women [81].

In agreement with all of the above, a number of works in murine models fed with high fat diets (HFD) (Table 2) supplemented with different pure flavanols (EGCG or proanthocyanidin dimer) or flavanol-rich foods (cocoa, green tea or grape seeds) have also shown a reduction in the inflammatory situation together with a diminished macrophage infiltration [79,80,82,83,84,85] (Table 1 and Table 2). At molecular level, decreased values of pro-inflammatory cytokines (TNF-α, IL-6 or MCP-1 by ~40–90%), and diminished pro-inflammatory enzymatic activities (iNOS, COX-2, adipose-specific phospholipase A2 by 80%, 55% and 53%, respectively) were associated to the downregulation of key proteins for the inflammatory process (NF-κB, MAPKs, TLR-4 and signal transducer and activator of transcription 3 [STAT3]), reduced macrophage infiltration in the adipose tissue, and a switch towards the less inflammatory M2-like phenotype macrophages [79,80,82,83,84,85]. Moreover, these outcomes have been related to a reduced endotoxemia by restoring the gut barrier [83], improved insulin signaling [80,82], as well as glucose and lipid metabolism [80,84], and redox status [84]. Benefits associated to a decreased inflammation (TNF-α, IL-6 by 25–60%, respectively) and macrophage infiltration (CD68, F4/80, by ~25% and 50%, respectively) have also been demonstrated in the pancreas [86] and aorta [87] after supplementing high fat diets with EGCG; these diets contributed to alleviate the condition of obesity by enhancing insulin sensitivity and reverting metabolic alterations [86,87]. Remarkably, flavanol (grape seed procyanidin and green tea polyphenol extract) supplementation to rats fed with high fat diets during pregnancy and lactation seem to be able to mitigate the immunity and inflammatory responses (macrophage infiltration) in the offspring, demonstrating an improved renal and adipose tissue function, lipid and glucose metabolisms [88,89].

In the metabolic syndrome, altered inflammatory and immune responses are also present [4] (Table 2). However, green tea polyphenols, including EGCG, large yellow tea and grape seed procyanidins have been reported to alleviate the inflammation and boost the immune system in animals fed with high fat and sucrose diets to induce the metabolic syndrome [90,91,92,93,94]. In addition, ameliorated glucose intolerance, insulin resistance and improved redox status and lipid metabolism were demonstrated [90,91,93,94]. Mechanistically, decreased levels of pro-inflammatory cytokines (TNF-α, IL-1β, IL-6, MCP-1, etc. by ~25–70%) induced by flavanols occur through the diminution of TLR4 and NF-κB values, as well as improved both redox status and immunity markers (CD11b, macrophage marker F4/80 by ~70% and 50%, respectively) [90,91,92,93,94]. In this regard, green tea polyphenols and a combination with procyanidins promoted the switch of immune cells towards a less inflammatory phenotype immune cells [90,92], and improved both thymus and spleen function by generating new T lymphocytes and their responsiveness [92].

**Figure 2:**
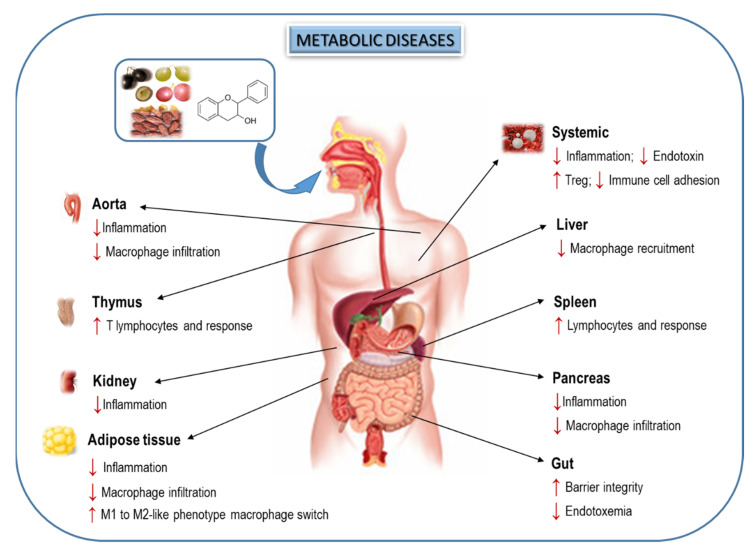
Flavanols and flavanol-rich foods exert beneficial effects related to inflammation and immune system on relevant metabolic diseases (diabetes, obesity and metabolic syndrome). The arrow indicates an increase (↑) or decrease (↓) in the level or activity of the different parameters analyzed.

Altogether, dietary flavanols could be suggested as promising agents to ameliorate the pro-inflammatory status and to counteract the metabolic disease-induced weakened immune response (Figure 2); however, more studies are needed to unravel the molecular mechanism of action of these natural compounds, especially to explain their regulation on the connection between inflammation and immune system. In this regard, the close relationship between flavonoids and GM could provide an additional potential mechanism to modulate host immunity and inflammation on metabolic diseases.

## 6. Interplay of Dietary Flavanols and Gut Microbiota in Metabolic Diseases

Numerous evidences have indicated the relevance of GM composition and function in metabolic diseases and the prominent role of diet in modulating GM. In this sense, flavanols can shape the microbial population to favor the increase of beneficial bacteria and inhibit the growth of pathogenic ones.

Several in vitro and animal studies have reported the prebiotic and antimicrobial effects of both isolated flavanols and flavanol-rich foods (mainly cocoa, green tea or grape seeds). In an in vitro study, Zhang et al. [95] showed that fermentation of EGCG, (+)-gallocatechin gallate (GCG) and 3-*O*-(3-*O*-methyl) gallate (EGCG3”Me) by human intestinal microbiota induced the growth of *Bifidobacterium* spp. and *Lactobacillus*/*Enterococcus* groups, and inhibited the growth of *Bacteroides-Prevotella*, *Clostridium histolyticum* and *Eubacterium–Clostridium* groups. Likewise, the in vitro fermentation of (+)-catechin increased the growth of the *Clostridium coccoides*–*Eubacterium rectale* group, *Bifidobacterium* spp. and *Escherichia coli*, and inhibited the growth of the *C. histolyticum* group. In contrast, (−)-epicatechin only was able to increase the growth of the *Clostridium coccoides–Eubacterium rectale* group [96]. In addition, the intake of flavanol-rich foods can also modulate the diversity and composition of GM in vivo. It has been shown that flavanol consumption can increase beneficial gut bacteria, such as *Lactobacillus and Bifidobacterium,* whereas reduce the number of pathogenic ones, such as *Clostridium perfringens* [66]. More importantly, cocoa supplementation (the most flavanol-rich food) in rats during 6 weeks was able to modify the microbiota composition and positively modulated the intestinal immune system, through changes in the colonic TLR pattern [97].

More importantly, flavanol intake has also been associated with a beneficial modulation of GM in metabolic diseases. Up to now, data regarding T2D are very scarce and only few studies have shown that supplementation with foods rich in flavanols, such as tea [98,99,100,101], cocoa [102] and grapes [103] could alleviate microbiota dysbiosis in T2D rodents. Among them, the last two works have also explored the impact of these flavanol-rich foods on immunity and inflammation (Table 3). In the first study [102], modifications in GM induced by the administration of a 10% cocoa-rich diet during 10 weeks were closely associated with an improved glucose homeostasis and intestinal integrity, as well as to a reduced inflammation in Zucker diabetic fatty (ZDF) rats. In particular, cocoa diet increased the abundance of acetate-producer bacteria, such as *Blautia*, and prevented the increase of lactate-producer bacteria (mainly *Enterococcus* and *Lactobacillus* genera) observed in diabetic animals. Likewise, cocoa supplementation increased the expression levels of ZO-1 and mucin glycoprotein above control values (100% and 50%, respectively). In addition, the cocoa-rich diet totally avoided the immune cell infiltration, the increase in IL-6 levels in the colonic mucosa of diabetic animals, and the decrease of TNF-α and MCP-1 levels by 50%, reducing thus the occurrence of metabolic endotoxemia. In the study by Tveter et al. [103], diabetic *db*/*db* mouse, which were supplemented with an extract of grape polyphenols for 4 weeks, showed an increased abundance of phylum Verrucomicrobia mainly due to a bloom in *Akkermansia muciniphila* at the expense of other taxa. *A. muciniphila* bloom has been associated with reduced inflammation and increased gut barrier integrity; however, in this study, grape supplementation did not change the intestinal gene expression of markers associated with metabolic endotoxemia (*Tjp1*, *Ocln*, *Muc2*, *Tnf-α*, *Il-6*, *inos*) or LPS serum levels. In contrast, the improved glucose homeostasis found in grape-supplemented *db*/*db* mice was related with a reduction of gut bacterial taxa associated with secondary BAs production. As a result, levels of primary BAs in serum were doubled in supplemented diabetic mice, leading to the FXR inhibition and the suppression of FXR-regulated genes required for biosynthesis of ceramides, which impairs glucose homeostasis.

In addition, flavanols can ameliorate some unfavorable changes in microbial composition caused by obesity or high-fat diets (HFD) (Table 3). It has been revealed that the long-term (13 weeks) infusion of green tea, oolong tea and black tea could modulate a wide range of intestinal microbes in high-fat-induced obese mice, contributing to the amelioration of their obesity [104]. Furthermore, the administration of EGCG3″Me for 8 weeks regulates the dysbiosis and maintains the balance of the microbial ecology in human flora-associated (HFA) mice fed with HFD [105]. Interestingly, this microbial reshape has been connected with the protective effects of flavanols against the intestinal barrier dysfunction induced by obesity. For example, the daily supplementation of grape pomace extract (GPE) in dietary-induced obese mice decreased the relative abundance of *Desulfovibrionaceae* and *Lactococcus* (linked with HFD) and increased the relative abundance of *Allobaculum* and *Roseburia,* which play an important role in gut health [106]. More importantly, the expression of several antimicrobial peptides, which were decreased in obese animals, was restored (Reg3γ) or even doubled (Lyz1) in obese mice supplemented with GPE, indicating an improvement in the gut barrier function. Furthermore, the gut dysbiosis induced by HFD may increase the intestinal permeability, resulting in elevated systemic LPS. However, it has been described that supplementation with three water extracts of green, oolong and black teas can suppress the production of LPS, most likely by the changes in the GM composition induced by the extracts [107].

Flavanols and flavanol-rich foods can also modulate gut microorganisms that may be related to gastrointestinal immunity and defense against inflammation. Accordingly, grape seed procyanidin extract (GSPE) supplementation prevented the increased in pro-inflammatory cytokines (TNF-α, IL-1β and IL-6) in the ileum of HFD-induced obese mice, restored the expression of tight junction proteins, and increased the proportion of CD4+, CD25+ and Foxp3+Treg in gut-associated lymphoid tissue [108]. As a result, GSPE improved intestinal barrier function and reduced LPS levels by 70% in the circulatory system of obese animals. Notably, all these beneficial effects were associated with changes in the composition of the GM induced by GSPE (mainly by decreasing the proportions of *Ruminococcaceae_UCG-005*, *Bacteroidales S24-7* and *Ruminococcus_1*). Similarly, a green tea polyphenol extract treatment was able to suppress the induction of TLR4 that, in turn, down-regulated the NF-κB signaling pathway, as well as the expression of inflammatory cytokines (TNF-α, IL-1β and IL-6) in intestinal tissues of canines fed with HFD [109]. More importantly, these outcomes were related to a decrease in the relative abundance of *Bacteroidetes* and *Fusobacteria* and to an increase in the relative abundance of *Firmicutes*. Similarly, the impact of GM could also affect inflammation in peripheral tissues. Accordingly, the supplementation with a catechin-rich green tea extract attenuated the gut dysbiosis in diet-induced obese mice, and totally prevented gut-derived endotoxin translocation, and consequent hepatic and adipose TLR4/NF-κB inflammation [110]. In addition, GSPE treatment in HFD-fed mice decreased systemic and metabolic tissue inflammation and totally avoided macrophage infiltration in epidydimal fat and liver tissues by modulating the gut microbial composition [111]. Notably, these beneficial effects of GSPE on inflammation were abolished when GM was depleted by an antibiotic treatment, proving the important role of the microbiota in these processes. Similar results were found in mice fed HFD that were supplemented with a tea-fermented extract (Pu-erh tea, PTE). PTE intake modulated GM in diet-induced obese mice and reduced serum LPS concentration and levels of hepatic pro-inflammatory cytokines by 80% [112]. Interestingly, the transplantation of feces from the PTE-treated animals reduced metabolic abnormalities in the obese recipient mice, demonstrating that the alteration of GM can mediate the beneficial metabolic effects of PTE. More recently, the restoration of GM in diet-induced obese mice by a grape extract (GE) administration has been associated with an altered BA pool in the serum. In particular, the abundance of *Akkermansia*, *Clostridium* and *Bifidobacterium* was negatively correlated with the concentrations of tauro-conjugated BAs, which are well-known FXR antagonists, indicating the potential role of these bacterial groups in the degradation of BAs [113]. In the same line, administration of EGCG to diet-induced obese mice enriched specific microbial (mainly *A. muciniphila*) and primary BAs levels, contributing thus to the metabolic benefits of EGCG [114]. It is interesting to mention that purified (−)-EGCG or (+)-catechin supplementation can also prevent hepatic inflammation in diet-induced obese mice, even though they exert differential prebiotic and antimicrobial activities [115]. Likewise, procyanidin B2 treatment ameliorated low-grade inflammation, decreased the levels of serum LPS by 50%, and protected against diet-induced obesity via the modulation of the GM in rabbits [116] (Table 3).

Finally, the two-way interaction between flavanols and GM could also contribute to the observed beneficial effects of flavanols in metabolic diseases. Indeed, flavanols are degraded by the microbiota into small bioactive phenolic acids increasing thus the bioavailability and bioactivity of these natural compounds. These phenolic metabolites derived from flavanol show anti-inflammatory activities and positively affect the immune system, therefore reducing the risk of metabolic disorders, as well as they improve the gut and overall host health [117].

**Figure 3:**
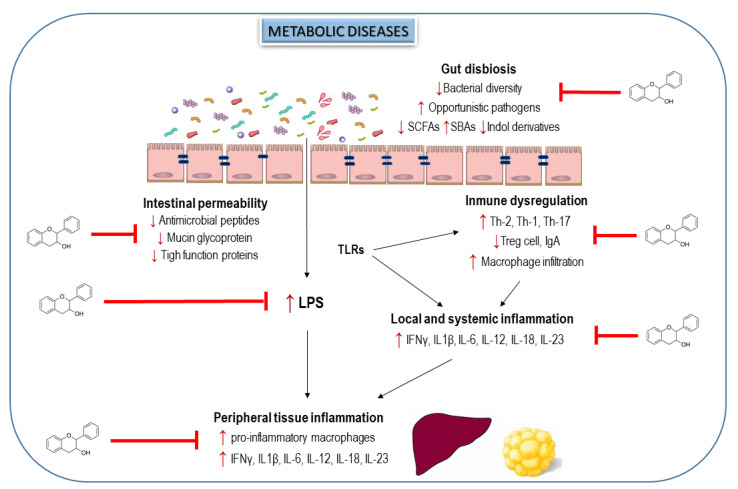
Effects of flavanols and flavanol-rich food on alterations in gut microbiota, immune system and inflammation during metabolic disorders. Up arrows indicate a rising trend and down arrows display a decreasing trend. Flavanols modulate gut dysbiosis, improve gut barrier function and the immune system and reduce serum endotoxin minimizing intestinal, hepatic and adipose inflammation.

Altogether, the compelling evidence from preclinical studies reveal that flavanols and flavanol-rich foods could modulate the gut dysbiosis in metabolic disorders and improve the gut barrier function, reducing thus the immune system dysregulation, as well as intestinal, hepatic and adipose inflammation (Figure 3). However, the absence of clinical human studies evaluating these beneficial effects limits the potential therapeutic utility of flavanols to prevent metabolic diseases by manipulating GM. In this sense, a very recent publication by Hodges et al. [118] describes an elegant protocol to develop clinical trials to evaluate the efficacy of flavanols in alleviating gut dysbiosis, intestinal permeability, metabolic endotoxemia and intestinal and systemic responses implicated on the inflammation in subjects with metabolic syndrome.

## 7. Conclusions and Future Perspectives

In view of all studies exposed, flavanols seem to play a prominent role on the protection against relevant metabolic diseases (diabetes, obesity and metabolic syndrome) by modulating inflammation, immune system and microbiota. Indeed, it seems that these natural compounds are able to modulate all these complicated connections to improve the general health status during these metabolic disorders. All these evidences would lead to propose these natural compounds as potential preventive tools useful for the nutritional management of the mentioned metabolic disorders. However, further studies are needed in order to ensure the efficacy of flavanols on the evaluated diseases, especially in humans where works studying the complex interplay among inflammation, immune system and microbiota exposed in this review are still very scarce; additionally, a deeper knowledge of the molecular mechanisms of these natural compounds on the immunity-inflammation-microbiota connections is mandatory. Thus, integration of all this information would allow the definition of optimal doses and duration of administration to finally obtain a beneficial effect in humans on major metabolic diseases, such as diabetes, obesity and metabolic syndrome.

## Figures and Tables

**Table 1:** Summary of the most relevant effects of flavanol and flavanol-rich product treatments on immunity, inflammation and metabolism in cultured cells ^a^.

Reference	Cellular Model	Flavanol/Food	Concentration	Duration	Main Outcomes
[68]	HAEC cells + 25 mM glucose	EGCG	1 µM	72 h	↓monocyte adhesion to HAEC, ↓NF-κB.
[69]	THP-1 cells (human monocytes) + 25 mM glucose	EC	5 µM	24 h	↓TNF-α, ↓NF-κB, ↓acetyl CBP/p300, ↓HDAC4, ↓H3K9ac/H3, ↓H3K4m2/H3, ↑H3K9me2/H3
[72]	PBMC (from 20 obese volunteers) induced with PMA	Red grape polyphenolic extract	1, 3, 5 µg/mL	24 h (co-treatment)	↓IL-21, ↓IL-1β, ↓IL-6, =IL-17, =TNF-α, ↑IL-10, =IFN-γ, =IL-4, =IL-2
[73]	Macrophages LPS-induced (U937 monocytes)	GPE	0, 30, 60, 100 µg/mL	4 h (GPE, 1 h + 100 ng/mL LPS, 3 h)	↓TNF-α, ↓IL-6, ↓IL-1β, ↓IL-8, ↓IP-10, ↓MCP-1, ↓COX-2, ↓JNK, ↓p38, ↓NF-κB, ↓cJun, ↓Elk-1, ↑IκBα
[73]	Adipocytes (abdominal WAT from obese volunteers) induced with macrophage-conditioned media	GPE	0, 30, 60, 100 µg/mL	4 h (GPE, 1 h + 100 ng/mL LPS, 3 h)	↓IL-6, ↓IL-8, ↓MCP-1, ↓IL-1β, ↓NF-κB, ↓glucose uptake in adipocytes
[74]	Human white differentiated adipocytes+RAW264 macrophages (co-culture)	Oligomerized grape seed polyphenols	10, 20 µg/mL	24 h	↓TNF-α, ↓MCP-1, ↓PAI-1, ↓ERK, ↓NF-κB, ↓ROS, =FFA release
[75].	Mouse 3T3L1 differentiated adipocytes + RAW264.7 macrophages (co-culture)	Theflavin-3,3′-digallate	0, 25, 50 µM	48 h	↓NO; ↓TNF-α, ↓IL-1β, ↓IL-6, ↓MCP-1, ↓iNOS, ↓CCR7, ↓CD86, ↓CD80, ↑IL-10, ↑CD206, ↑CD163, ↑arginase-1 ↑PPAR-γ, ↓p-IKK, ↓p-IκB, ↓p-p65-NF-κB, ↓COX-2, ↓p-STAT3, ↓ROS, ↓TG, ↓NEFA, ↓FAS, ↓glycerol, ↑ adiponectin, ↑AMPK.
[76]	RAW264.7 macrophages (1 µg/mL LPS)	CAE	31–500 µg/mL	24 h	↓NO, ↓PGE2, ↓TNF-α, ↓MCP-1, ↓IL-6, ↓ROS.
[76]	Mouse 3T3L1 adipocytes (conditioned media)	CAE	31–500 µg/mL	24 h	↓TNF-α, ↓MCP-1, ↓IL-6, ↓TG, ↓lipid content, ↑glycerol release, ↑lipase activity, ↑adiponectin, ↑mitochondrial function and content, ↑PGC-1α, ↑UCP-1, ↑glucose uptake, ↑GLUT-4 translocation, ↑IR, ↑PI3K, ↑AKT.
[77]	PBMC (12 lean + 12 obese, sex-matched)	EGCG	20 µM	24 h	↓NF-κB, ↑Fox3p-positive Tregs, ↑IL-10, ↑HDCA2 in Tregs and HDAC activity.
[78]	PBMC (obeses receiving grape powder for 9 weeks) LPS-stimulated (1 µg/L)	Grape powder	23 g (2×/day, 3.79 mg PP/day)	24, 48 and 72 h	↑IL-1β, ↑IL-6, =IL-8.
[79]	Mouse 3T3L1 differentiated adipocytes + RAW264.7 macrophages (co-culture)	GTE+ citrus PMFs+ lychee polyphenols	10–100 µg/mL	24 h	↓IL-6, ↓IL-1β, ↓iNOS, ↑p21, ↑p53, ↑AMPK, ↑cyclinE1, ↓CDK2, ↓proliferation, ↓differentiation, ↓C/EBPs, ↓PPARγ.
[80]	Mouse 3T3L1 differentiated adipocytes (5 ng/mL TNF-α, co-treatment)	GC-(4→8)-GCG	10, 20 µg/mL	24 h	↓MCP-1, ↓IL-6, ↓COX-2, ↓TG, ↓lipid content, ↓epididymal, ↓PPARγ, ↓SREBP-1c, ↓C/EBPα, ↓p-JNK, =ERK, ↓p-p38↓p-STAT3, ↓p-IκB, ↑IκB, ↓p-p65-NF-κB

^a^ The arrow indicates an increase (↑) or decrease (↓) in the level or activity of the different parameters analyzed, “=” symbol designates unchanged parameters. AKT/PKB: protein kinase B; AMPK: adenine monophosphate activated protein kinase; CAE: cocoa shell aqueous phenolic extract; CCR7: C-C chemokine receptor type 7; C/EBPα: CCAAT/enhancer-binding protein alpha; CDK2: cyclin-dependent kinase 2; COX-2: cyclooxygenase-2; EC: epicatechin; EGCG: epigallocatechin gallate; Elk1: ETS Like-1 protein; ERK: extracellular signal-regulated kinase; FAS: fatty acid synthase; FFA: free fatty acid; Fox3p: forkhead box p3 transcription factor; GC-(4→8)-GCG: gallocatechin-(4→8)-gallocatechin-3-*O*-gallate; GLUT: glucose transporter; GPE: polyphenol-rich grape powder extract; GTE: green tea extract; HAEC: human aortic endothelial cells; HDCA: histone deacetylase; H3K9ac: histone H3 lysine 9 acetylation; H3K4me2: histone H3 lysine 4 dimethylation; H3K9me2: histone H3 lysine 9 dimethylation; IFN-γ: interferon-γ; IκB: inhibitor κB; IKK: IκB kinase; IL: interleukin; iNOS: inducible nitric oxide synthase; IP-10: interferon-γ inducible protein-10; IR: insulin receptor; JNK: c-Jun NH2-terminal kinase; LPS: lipopolysaccharide; MAPK: mitogen activated protein kinases; MCP-1: monocyte chemotactic protein-1; NEFA: non-esterified fatty acid; NF-κB: nuclear factor-kappa B; NO: nitric oxide; p38: p38-MAPK; PAI-1: plasminogen activator inhibitor-1; PBMC: peripheral blood mononuclear cells; PGC-1α: Peroxisome proliferator-activated receptor-gamma coactivator-1α; PGE2: protastglandin E2; PI3K: phosphatidylinositol 3-kinase; PMA: phorbol myristate acetate; PMFs: polymethoxyflavones; PP: polyphenol; PPAR: Peroxisome proliferator activated receptor; ROS: reactive oxygen species; SREBP: sterol regulatory element-binding protein; STAT: signal transducer and activator of transcription; TG: triglycerides; TNF-α: tumor necrosis factor α; Treg: regulatory T cells; UCP: uncoupling protein; WAT: white adipose tissue.

**Table 2:** Summary of the most relevant effects of flavanol and flavanol-rich product administrations on immunity, inflammation and metabolism during diabetes, obesity and metabolic syndrome in animal and human studies ^a.^

Reference	Experimental Model	Flavanol/Food	Dose	Duration	Main Outcomes
Diabetes					
[68]	*db*/*db* mice	EGCG	0.1% (of diet)	8 weeks	↓monocyte adhesion to endothelial cells, ↓MCP-1, ↓KC, ↓ICAM-1, ↓VCAM-1, ↓NF-κB, ↓BP, ↓Cho, ↓TG.
[70]	GK rats (peripheral leukocytes)	EGCG	0.1%, 0.2% and 0.5%	25 weeks	↓mRNA TNF-α, ↓IFN-γ, ↓IL-1β, ↓IL-6, ↓IL-18, ↓MCP-1, ↓CD116, ↓S100A6, ↓8-OHdG, ↓MDA, =CD18, ↓BW, =GLU, ↓INS, ↓TG, =ALT, =AST.
[71]	GK rats (mesenteric adipose tissue)	EGCG	0.1%, 0.2% and 0.5%	25 weeks	=HbA1c, =CD-18, ↓IL-1β, ↓TNF-α, ↓IL-6, ↓IL-12, ↓IL-18, ↓MCP-1, ↓resistin, ↓PAI-1.
Obesity					
[78]	RCDB-cross over (24 obese, 20–60 y, 16 ♀ + 8 ♂)	Grape powder	23 g (2×/day, 3.79 mg PP/day)	9 weeks	=IL-1β, =IL-6, =IL-8, =TNF-α, =sICAM-1, =sVCAM-1, =CRP, =leptin, =serum amyloid A, =BMI, =BW, =antioxidant status (ORAC, oxLDL), ↓large LDL-Cho, ↓LDL particles, =ALT, =AST, =alkaline phosphatase.
[81]	CSIS (8 lean ♀ + 10 obese ♀, 27–48 y)	Green tea extract	1009.6 mg (450.7 mg EGCG)	8 weeks	↑telomere length in leukocytes in lean and obese participants, =BW, =BMI, =GLU, =alkaline phosphatase, =HDL-Cho, =TG, =AST, ↓LDL-Cho, ↓total Cho, ↓ALT, ↓GGT.
[83]	HFD fed mice (60% Kcal from fat)	Cocoa	8% (of diet)	18 weeks	↓TNF-α, ↓IL-6, ↓iNOS, ↓Emr-1, ↓NF-κB, ↓arachidonic acid, ↓COX-2, ↓phospholipase A2, ↓plasmatic endotoxin, ↓GLP-2, =BW, =food intake, =fat weight, =GLU, ↓TG, ↓FFA, ↓INS, ↓HOMA-IR.
[82]	HFD fed rats (60% Kcal from fat)	EGCG	3.2 g/Kg (of diet)	16 weeks	↓TLR4, ↓TRAF6, ↓p-IκB, ↓p-NF-κB, ↓TNF-α, ↓IL-6, ↓macrophage infiltration, ↓CD68, ↓BW, ↓epididymal adipose weight, =food intake, =GLU, ↓FFA, ↓INS, ↓HOMA-IR, ↓p-IRS-1, =IRS-1, ↑PI3K (p85), ↑GLUT4.
[79]	HFD fed mice (45% Kcal from fat)	GTE+ citrus PMFs+ lychee polyphenols	0.1–0.5% (of diet)	16 weeks	↓MCP-1, ↓IL-6, ↓macrophage infiltration (↓F4/80, ↓CD11b), ↑CD163, ↑IL-10.
[80]	HFD fed mice (60% Kcal from fat)	GC-(4→8)-GCG	40 and 80 mg/Kg	8 weeks	↓MCP-1, ↓IL-6, ↓TNF-α, ↓F4/80, ↓CD11b, ↓BW, =food intake, ↓GLU, ↑glucose tolerance, ↑insulin sensitivity, ↓TG, ↓liver weight, ↓hepatic lipid content, ↓epididymal, inguinal and perirenal fat, ↓adipocyte size, ↑adiponectin, ↓leptin, ↓PPARγ, ↓SREBP-1c, ↓C/EBPα, ↓p-STAT3, ↓p-IκB, ↑IκB, ↓p-p65-NF-κB
[84]	HFD fed mice (47% Kcal from fat)	Defatted Chardonnay grape seed flour	10% (of diet)	5 weeks	↓*Tnf*, ↓*Tril*, ↓*Il7r*, ↓*Adam 8*, ↓*Il1rn*, ↓*H2-M2*, ↓*Lbp*, ↓iNOS, ↓*Otop1*, ↓TLR4, ↓*Igsf6*, ↓*Cnr2*, ↓*Msr1*, ↓*Ncf4*, ↓*Mmp19*, ↓*CD68*, ↑PPARγ, ↓*Cebpb*, ↓BW, ↓liver weight, ↓GLU, ↓epididymal adipose tissue weight, ↓leptin.
[85]	HFD fed rats (60% Kcal from fat)	Grape-seed procyanidin extract	1–2 mg/animal	30 days	↓TNF-α, ↓CRP, ↓IL-6 (serum and adipose tissue), ↓BW, ↓adiponectin, ↓Emr1, ↓macrophage infiltration, ↓NF-κB (liver), ↓TNF-α (liver).
[86]	HFD fed rats (60% Kcal from fat)	EGCG	3.2 g/Kg (of diet)	16 weeks	↓TNF-α, ↓IL-6, ↓macrophage infiltration, ↓CD68, =TLR4, =TRAF6, ↓BW, =food intake, ↓INS, =GLU, ↓HOMA-IR, ↓FFA
[87]	HFD fed mice (60% Kcal from fat)	EGCG	50 mg/Kg/day	10 weeks	↓macrophage infiltration, ↓F4/80, ↓BW, =GLU, ↓leptin, ↓INS, ↓QUICKI, ↓F4/80 (adipocytes), ↑p-eNOS, ↑p-IRS-1, ↑p-AKT.
[88]	HFD fed rats (60% Kcal from fat)	Grape seed procyanidin extract	25 mg/Kg bw	30 days	In the offspring: ↓MCP-1, ↓*Ccl3*, ↓*Cl11*, ↓*Ccl12*, ↓p*hospholipase A2*, ↑complement factor I, ↑complement components, ↑adiposity index ↑number of cells in epidydimal adipose tissue, =GLU, =INS, =leptin, =adiponectin, =TG, =total Cho, =FFA, ↓glycerol.
[89]	HFD fed rats (45% Kcal from fat)	Green tea extract	0.12–0.24% (of diet)	45 weeks	In the offspring: ↓TNF-α, ↓COX-2, ↓PAI-1, ↓macrophage infiltration (CD-68), ↓TGF-β, ↓fibrosis, ↓BW, ↓GLU, ↓TG.
Metabolic syndrome					
[90]	Cafeteria diet fed rats	Green tea	500 mg/Kg bw/day	12 weeks	Lymphocytes: ↓IL-2, ↓IL-6, ↓IL-1β, ↓TNF-α, ↓TLR4, ↑IL-10, =IFNγ, =T-bet, =GATA-3, =Foxp3, ↑IRF4, ↓cell proliferation, ↓hexokinase, ↓G6PDH, ↓ROS, ↑MnSOD, ↑CuSOD, ↑GPx, ↑GR, ↑Nrf2, =CAT, ↓BW, ↓FFA, =leptin, ↑adiponectin, ↓glucose intolerance, ↑insulin sensitivity.
[91]	Cafeteria diet fed rats	Green tea	500 mg/Kg bw/day	12 weeks	Neutrophils: ↑migration capacity, =phagocytic capacity, ↓TNF-α, ↓IL-6, =IL-1β, ↓TLR4, ↓CD11b, ↓IKK, =NF-κB, ↓MPO, ↑hydrogen peroxide, ↑hypochlorous acid, ↑superoxide anion, ↓CAT, =GPx, =GR, =GSH, =GSSG, ↑GSH/GGSG, =hexoquinase, ↓Nrf2, =leptin receptor B, ↓glucose intolerance.
[92]	Cafeteria diet fed rats	Grape seed procyanidin extract	25 mg/Kg bw	13 weeks (3 weeks supplementation)	Adipose tissue: ↓F4/80, ↓TNF-α, ↓IL-6, ↑Foxp3, ↑IL-10, ↓iNOS. =BW. Serum: =MCP-1, ↓complement factor 3, =leptin, =adiponectin. Thymocytes (thymus and spleen): =IL-6, ↑IL-10, ↓F4/80, =TNF-α.
[93]	HFD fed mice (45% Kcal from fat)	Large yellow tea	0.5 and 2.5% (*w*/*w*)	12 weeks	↓number of adipocytes, ↓TNF-α, ↓MCP-1, ↓IFNγ, ↓IL-6, =IL-1β, =IL-4, =IL-10, ↓macrophage infiltration, ↓BW, ↓liver weight, ↓adipose tissue weight, ↓INS, ↓GLU, ↑glucose tolerance, ↑insulin sensitivity, ↓TC, ↓TG, ↓LDL, ↓HDL, ↑adiponectin.

^a^ The arrow indicates an increase (↑) or decrease (↓) in the level or activity of the different parameters analyzed, “=” symbol designates unchanged parameters. *Adam 8:* a disintegrin and metallopeptidase domain 8; AKT/PKB: protein kinase B; ALT: alanine aminotransferase; AST: aspartate aminotransferase; BMI: body mass index; BP: blood pressure; BW: body weight; CAT: catalase; *Ccl:* chemokine (C-C motif) ligand; C/EBPα:CCAAT/enhancer-binding protein alpha; Cho: cholesterol; *Cnr2:* cannabinoid receptor 2; COX-2: cyclooxygenase-2; CRP: C-reactive protein; CSIS: cross-sectional interventional study; EGCG: epigallocatechin gallate; Emr1 or F4/80: EGF-like module-containing mucin-like hormone receptor-like 1; eNOS: endothelial nitric oxide synthase; FFA: free fatty acid; Fox3p: forkhead box p3 transcription factor; GATA-3: GATA binding protein 3; GC-(4→8)-GCG: gallocatechin-(4→8)-gallocatechin-3-*O*-gallate; GGT: gamma-glutamyl transferase.; GLP2: glucagon-like peptide; GLU: glycemia; GLUT: glucose transporter; G6PDH: Glucose-6-phosphate dehydrogenase; GPx: glutathione peroxidase; GR: glutathione reductase; GSH: reduced glutathione; GSSG: oxidized glutathione; GTE: green tea extract; HbA1c: glycated hemoglobin; HDL: high-density lipoprotein; HFD: high-fat diet; *H2-M2*: histocompatibility 2, M region locus 2; HOMA-IR: homeostasis model assessment of insulin resistance; ICAM-1: intercellular adhesion molecule-1; IFN-γ: interferon-γ; *Igsf6*: immunoglobulin superfamily, member 6; IκB: inhibitor κB; IKK: IκB kinase; IL: interleukin; *Il7r:* interleukin 7 receptor; *Il1rn:* interleukin 1 receptor antagonist; iNOS: inducible nitric oxide synthase; INS: insulinemia; IRF4: interferon regulatory factor 4; IRS: insulin receptor substrate; KC: mice chemokine most closely related to IL-8; *Lbp*: lipopolysaccharide binding protein; LDL: low-density lipoprotein; MCP-1: monocyte chemotactic protein-1; MDA: malondialdehyde; *Mmp19:* matrix metallopeptidase 19; MPO: myeloperoxidase; *Msr1*: macrophage scavenger receptor 1; *Ncf4*: neutrophil cytosolic factor 4; NF-κB: nuclear factor kappa B; NRF2: nuclear factor erythroid 2; 8-OHdG: 8-hydroxy-2′ deoxyguanosine; ORAC: oxygen radical absorbance capacity; *Otop1*: otopetrin 1; oxLDL: oxidized low-density lipoprotein; PAI-1: plasminogen activator inhibitor-1; PI3K: phosphatidylinositol 3-kinase; PMFs: polymethoxyflavones; PP: polyphenol; PPAR: Peroxisome proliferator activated receptor; QUICKI: quantitative insulin sensitivity check index; RCDB: Randomized Controlled Doble-Blind; ROS: reactive oxygen species; S100A6: S100 calcium binding protein A6; SOD: superoxide dismutase; SREBP: sterol regulatory element-binding protein; STAT: signal transducer and activator of transcription; T-bet: T-box expressed in T cells; TG: triglycerides; TGF-β: transforming growth factor-β; TLR: toll-like receptor; TNF-α: tumor necrosis factor α; TRAF6: TNF Receptor Associated Factor 6; *Tril:* TLR4 interactor with leucine-rich repeats; VCAM-1: vascular adhesion molecule-1.

**Table 3:** Summary of the most relevant effects of flavanol and flavanol-rich products administration on metabolism, immunity, inflammation and gut microbiota during diabetes and obesity ^a^.

Reference	Experimental Model	Treatment	Dose	Time (Weeks)	Metabolic Outcomes	Immunity, Inflammation and Gut Microbiota Outcomes
[108]	Zucker diabetic rats	Cocoa rich diet	10% (of diet)	10	↓BW, ↓GLU, ↓INS, ↓HbA1c, ↓HOMA-IR, ↑HOMA-B, ↓LDL	
[103]	*db*/*db* obese mice	Grape polyphenol diet	10% (of diet)	10	=BW, ↓GLU, ↑glucose tolerance	=(ZO-1, Occludin, Mucin 2 and serum LPS); =(TNFα, Il-6 and iNOS in ileum); ↑CA and TCA (PBAs) and ↓SBAs serum levels. ↑(*Akkermansia, Blautia, Clostridium);* ↓(*Anaeroplasma, Ruminococcus, Butyricicoccus Dehalobacterium,, Streptococcus, Dorea, Lactococcus, Oscillospira)*
[106]	C57BL/6J mice fed with HFD induced obesity	Grape pomace extract	8.2 (g/Kg bw)	8	=BW, ↓GLU, ↓INS, ↑glucose tolerance, ↓NEFAs, = Cho, = TG	=ZO-1, ↑(Occludin, Reg3γ, Lyz1);↓(Integrin alpha X, LBP, MCP1 and macrophages in adipose tissue); ↑(*Allobaculum, Roseburia*); ↓(*Desulfovibrio, Clostridium sensu stricto, Lactococcus*)
[107]	C57BL/6J mice fed with HFD induced obesity	Green, oolong and black tea water extracts	1% (*w*/*v*)	28	↓BW, ↓INS, ↑glucose tolerance, ↓Cho, = TG	↓(LPS and IL-6 in plasma); ↑(*Lachnospiracea, Ruminococcacea*) ↓ (*Rikenellaceae, Desulfovibrionaceae*)
[108]	Wistar rats fed with HFD induced obesity	Grape seed procyanidin extract	200 (mg/Kg bw)	13	↓BW, = GLU ↓Cho, ↓TG, ↓LDL. ↓HDL	↑(Occludin, ZO-1); ↓Gut permeability; ↓LPS in serum; ↓(TNF-α, IL-1β and IL-6 in ileum); ↑(CD4^+^, CD25^+^ Treg in GALT) ↑(*Butyricicoccus, Oscillospira Lachnospiraceae, Ruminococcaceae*) ↓(*Ruminococcaceae_UCG-005, Bacteroidales S24-7* and *Ruminococcus_1*)
[109]	Canines fed with HFD induced obesity	Green tea polyphenols	1.92% (g/Kg diet)	18	↓BW	↓(TNF-α, IL-1β and IL-6 in ileum), ↑TLR4 signaling pathway ↑(*Acidaminococcus, Succinivibrio* and *Citrobacter*) ↓(*Bacteroides, Fusobacterium* and *Anaerobiospirillum*)
[110]	C57BL/6J mice fed with HFD induced obesity	Catechin-rich green tea extract	2% (of diet)	8	↓BW, ↓GLU, ↓INS, ↓HOMA-IR ↓NEFAs, ↓Cho, ↓TG,	↑(Occludin, ZO-1); ↓LPS in serum; ↓(TNFα, iNOS, MCP-1), ↓(CD68m TLR4, MyD88) and = CD14 in epidydimal fat; ↓(TNFα and iNOS), ↓(CD14, TLR4) and = MD2 in ileum ↑(*Akkermansia, Butyrivibrion, Bifidobacterium*); ↓(*Lactobacillus, Ruminococcus*)
[111]	C57BL/6 mice fed with HFD induced obesity	Grape seed procyanidin extract	300 (mg/Kg bw)	7	=BW, ↑glucose tolerance, ↑insulin sensitivity	↓(TNFα, IL-6 and MCP-1) in plasma; ↓(F4/80, CD68 and MCP-1) in epidydimal fat and liver tissues ↑(*Clostridium XIVa, Escherichia/Shigella, Blautia, Flavonifractor, Arthrobacter, Roseburia spp* and *Roseburia inulinivorans*); ↓(*Lactococcus and Bacteroides*)
[112]	C57BL/6N mice fed with HFD induced obesity	Pu-erh tea extract	tea	0.4% (w/v)	↓BW, ↓GLU, ↑glucose tolerance, ↓Cho, ↓TG, ↓LDL, ↑HDL	↑(Occludin, ZO-1);↓LPS in serum; ↓(TNF-α, IL-1β and IL-6 in liver), ↑(*Anaerotruncus, Alistipes, Odoribacter, Akkermansia, Blautia, Bacteroides, Parabacteroides* and *Roseburia*); ↓(*Bilophila, Leuconostoc, Allobaculum*)
[113]	C57BL/6Cnc mice fed with HFD induced obesity	Grape extract	1% (*w*/*v*)	13	↓BW, ↓GLU, ↑glucose tolerance, ↑insulin sensitivity, ↓Cho	↓LPS in serum; ↓(TNF-α, IL-6) in serum; ↓(TNFα, IL-6 and MCP-1) in epidydimal fat and liver tissues. ↑ratio of conjugated/free BA , ↑ratio of secondary/primary BA↑(*Bifidobacteria, Akkermansia* and *Clostridia*) ;↓(*Bacteroides* and *Desulfovibrio*)
[114]	C57BL/6 mice fed with WD	EGCG	100 (mg/Kg bw)	8	↓BW, = GLU, ↑insulin sensitivity, ↓Cho, ↓TG	↓LPS in serum; ↓(F4/80, CD36) in adipose and liver; ↓serum BAs, ↑(*Enterococcaceae* and *Verrucomicrobiaceae* -mainly *A. muciniphila*-). ↓(*Lachnospiraceae, Desulfovibrionaceae, Bacteroidaceae, Prevotellaceae, Rikenellaceae* and *Deferribacteraceae*)
[115]	C57BL/6J mice fed with HFD induced obesity	EGCG	0.3% (*w*/*w*)	8	↓BW, ↓GLU, ↓INS, ↓HOMA-IR, ↓Cho	↑(Claudin-1, Occludin and ZO-1); ↓LPS in serum; ↓(TNFα, iNOS, MCP-1) and ↓(TLR4, MyD88) in liber; ↓TNFα in intestine. ↑(*Ruminococcaceae UBA1819 and Parasutterella*); ↓(*Ruminiclostridium, Clostridium, Blautia, Roseburia, Acetatifactor, Lachnoclostridium, Lachnospiraceae UCG-006*)
[115]	C57BL/6J mice fed with HFD induced obesity	Catechin	0.3% (*w*/*w*)	8	↓BW, ↓INS, ↓HOMA-IR, ↓Cho	↑(Claudin-1, Occludin and ZO-1); ↓LPS in serum; ↓(iNOS, MCP-1) and ↓(TLR4, MyD88) in liber; ↓TNFα in intestine. ↑(*Ruminiclostridium 9, Oscillibacter*); ↓(*Ruminiclostridium, Clostridium, Blautia, Roseburia*
[116]	New Zealand white rabbits fed with HFD induced obesity	Procyanidin B2	150 (mg/Kg bw)	12	↓BW, ↓INS ↓Cho, ↓TG, ↓LDL, ↑HDL	↓LPS in serum; ↑(*Ruminococcus, Bacteroidetes. Akkermansia*); ↓(*Allobaculum*)

^a^ The arrow indicates an increase (↑) or decrease (↓) in the level or activity of the different parameters analyzed, “=” symbol designates unchanged parameters. BW: body weight; CA: cholic acid; Cho: Cholesterol; EGCG: epigallocatechin-3-gallate; GALT: gut-associated lymphoid tissue; GLU: glycemia; HbA1c: hemoglobin glycosylated; HDL: high density lipoprotein; HFD: high fat diet; HOMA-B: homeostasis model assessment of beta cell function; HOMA-IR: homeostasis model assessment of insulin resistance; INS: insulinemia; LPS: lipopolysaccharide; LBP: lipopolysaccharide binding protein; LDL: low density lipoprotein; Lyz1: lysozyme C-1; MCP-1: monocyte chemoattractant protein-1; NEFAs: non-esterified fatty acid levels; iNOS: inducible nitric oxide synthase; PBAs: primary bile acids; Reg3γ: regenerating islet-derived protein 3 gamma; SBAs: secondary bile acids; TCA: taurocholic acid; TG: triglycerides; TNF-α: tumor necrosis factor α; TLR4: toll-like receptor 4; Tregs: T regulatory cells; WD: Western diet; ZO-1: zonula ocludens-1.
